# Peril of the Shallows?: Elevated Arsenic in Kelp Supplements

**Published:** 2007-04

**Authors:** Cynthia Washam

Kelp, widely consumed in Asian countries, is a growing part of the U.S. supplement market. It generally is marketed as a concentrated source of iodine and other essential minerals. Because kelp is a nutritional supplement and not a drug, the FDA does not require manufacturers to demonstrate safety or efficacy. Now researchers at the University of California, Davis, report the case of a woman who received toxic doses of arsenic from kelp supplements **[*EHP* 115:606–608; Amster et al.]**.

Arsenic occurs naturally in some soils, and can contaminate bodies of water. The metalloid concentrates in fish that eat arsenic-rich algae and can also be found in plants that absorb it from the soil or water in which they are grown. Human exposure typically comes from diet, contaminated drinking water, or occupational exposures, as in smelters; people ingest an average of 40 μg per day.

The researchers investigated kelp supplements after a 54-year-old woman taking the pills was referred to the university’s occupational medicine clinic. The patient had started taking kelp to treat minor memory loss and fatigue. She initially took the dose recommended on the bottle, then doubled it when her symptoms failed to improve. She took kelp for one year, during which her fatigue worsened to the point that she had to switch from full- to part-time work. She also experienced rash, diarrhea, vomiting, severe headaches, and hair loss.

A urine test revealed an arsenic concentration of 83.6 μg/g creatinine. Creatinine, a muscle metabolite, is excreted at a relatively constant rate and is used for reporting urinary biomarkers, as an adjustment for the high variability of urine dilution. A normal arsenic concentration is less than 50 μg/g creatinine.

The researchers sent the patient’s kelp supplement, along with several other brands purchased from local health food stores, to be tested at a state lab after ruling out occupational, dietary, and drinking-water exposure. Only one of the nine samples tested contained no detectable arsenic. Concentrations among the other eight ranged from 1.59 ppm to 65.5 ppm by dry weight. Samples taken from three batches of the patient’s brand had concentrations of 1.59, 2.28, and 34.8 ppm. The FDA tolerance level for arsenic is 2 ppm.

Three weeks after she abandoned kelp, the woman resumed full-time work. Her urine arsenic concentration dropped more than a third in two months and was undetectable after another two months. Eventually all her syptoms resolved.

Past studies have shown that many herbal remedies are contaminated with potential toxicants including mercury and lead. To prevent more inadvertent poisonings, the authors recommend that manufacturers be required to prove safety before marketing their products.

## Figures and Tables

**Figure:**
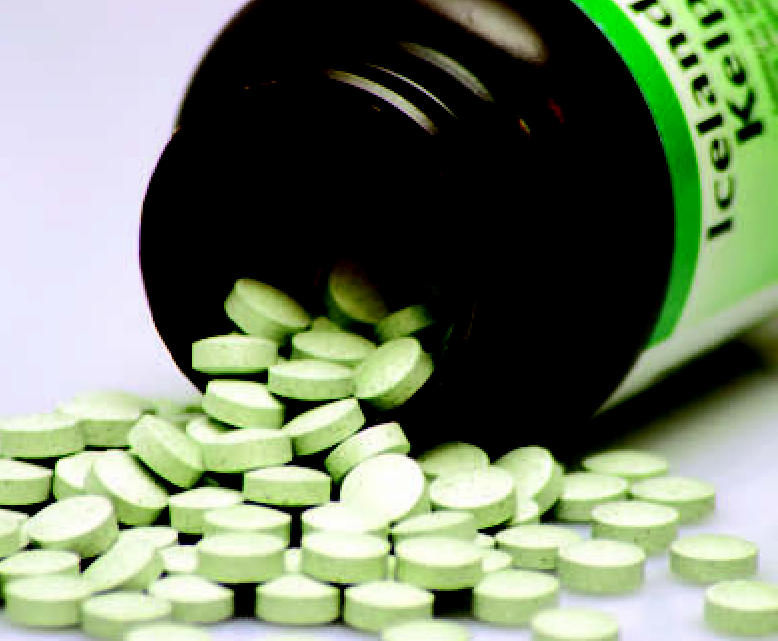
Herbal loophole As a dietary supplement, kelp is exempt from drug safety testing.

